# Intraoperative mean arterial pressure and acute kidney injury after robot-assisted laparoscopic prostatectomy: a retrospective study

**DOI:** 10.1038/s41598-023-30506-1

**Published:** 2023-02-27

**Authors:** Tae Lim Kim, Namo Kim, Hye Jung Shin, Matthew R. Cho, Hae Ri Park, So Yeon Kim

**Affiliations:** 1 Department of Anesthesiology and Pain Medicine, Anesthesia and Pain Research Institute, Yonsei University College of Medicine, 50-1 Yonsei-ro, Seodaemun-gu, Seoul, 03722 Republic of Korea; 2 Biostatistics Collaboration Unit, Yonsei University College of Medicine, Seoul, Republic of Korea

**Keywords:** Risk factors, Acute kidney injury, Prostate cancer, Medical research

## Abstract

Intraoperative hemodynamics can affect postoperative kidney function. We aimed to investigate the effect of intraoperative mean arterial pressure (MAP) as well as other risk factors on the occurrence of acute kidney injury (AKI) after robot-assisted laparoscopic prostatectomy (RALP). We retrospectively evaluated the medical records of 750 patients who underwent RALP. The average real variability (ARV)-MAP, standard deviation (SD)-MAP, time-weighted average (TWA)-MAP, area under threshold (AUT)-65 mmHg, and area above threshold (AAT)-120 mmHg were calculated using MAPs collected within a 10-s interval. Eighteen (2.4%) patients developed postoperative AKI. There were some univariable associations between TWA-MAP, AUT-65 mmHg, and AKI occurrence; however, multivariable analysis found no association. Alternatively, American Society of Anesthesiologists physical status ≥ III and the low intraoperative urine output were independently associated with AKI occurrence. Moreover, none of the five MAP parameters could predict postoperative AKI, with the area under the receiver operating characteristic curve values for ARV-MAP, SD-MAP, TWA-MAP, AUT-65 mmHg, and AAT-120 mmHg being 0.561 (95% confidence interval [CI], 0.424–0.697), 0.561 (95% CI, 0.417–0.704), 0.584 (95% CI, 0.458–0.709), 0.590 (95% CI, 0.462–0.718), and 0.626 (95% CI, 0.499–0.753), respectively. Therefore, intraoperative MAP changes may not be a determining factor for AKI after RALP.

## Introduction

Robot-assisted laparoscopic prostatectomy (RALP) is preferred to open prostatectomy since it allows minimal invasion, better short-term outcomes, and improved functional results^1,2^. However, pneumoperitoneum during RALP can induce direct compression of the renal vasculature, ureter, and kidney, which causes a reduction in the renal blood flow and glomerular filtration rate as well as oliguria^3,4^. The incidence of acute kidney injury (AKI) after RALP has been reported to be significantly lower than that after open radical prostatectomy^5^; however, AKI still occurs in approximately 5% of patients undergoing RALP^5,6^. Moreover, an acute increase in serum creatinine (SCr) levels after RALP usually occurs on the operation day^7,8^. Patients who develop AKI on the operation day have been reported to present reduced renal function at 12 months after RALP^8^. Therefore, careful perioperative management may be crucial for preventing AKI.

The steep Trendelenburg position with carbon dioxide (CO_2_) insufflation is necessary for optimizing surgical exposure during RALP. It results in remarkable hemodynamic alterations, including a > 30% increase in the mean arterial pressure (MAP)^9–11^. Moreover, there is a considerable abrupt decrease in MAP after resuming a supine position with CO_2_ desufflation^11,12^. Intraoperative blood pressure (BP) variability has an independent positive correlation with the risk of AKI after non-cardiac surgery^13^. Additionally, intraoperative low MAP is an established risk factor for AKI after non-cardiac surgery^13–17^. Therefore, abrupt changes in MAP during RALP can affect postoperative kidney function. Moreover, patients undergoing RALP are mostly older adults with various comorbidities, including hypertension and diabetes mellitus^5,6,8,12^, which may further increase BP variability and the intraoperative risk of hypotension^13,18^. However, the relationship between intraoperative MAP changes and postoperative AKI in patients undergoing RALP remains unclear. Therefore, this retrospective study aimed to investigate the association of intraoperative MAP (MAP variability, hypotension, and hypertension) with AKI occurrence after RALP. Additionally, we aimed to investigate the association between AKI following RALP and other factors.

## Results

### Patient characteristics

**Figure 1:**
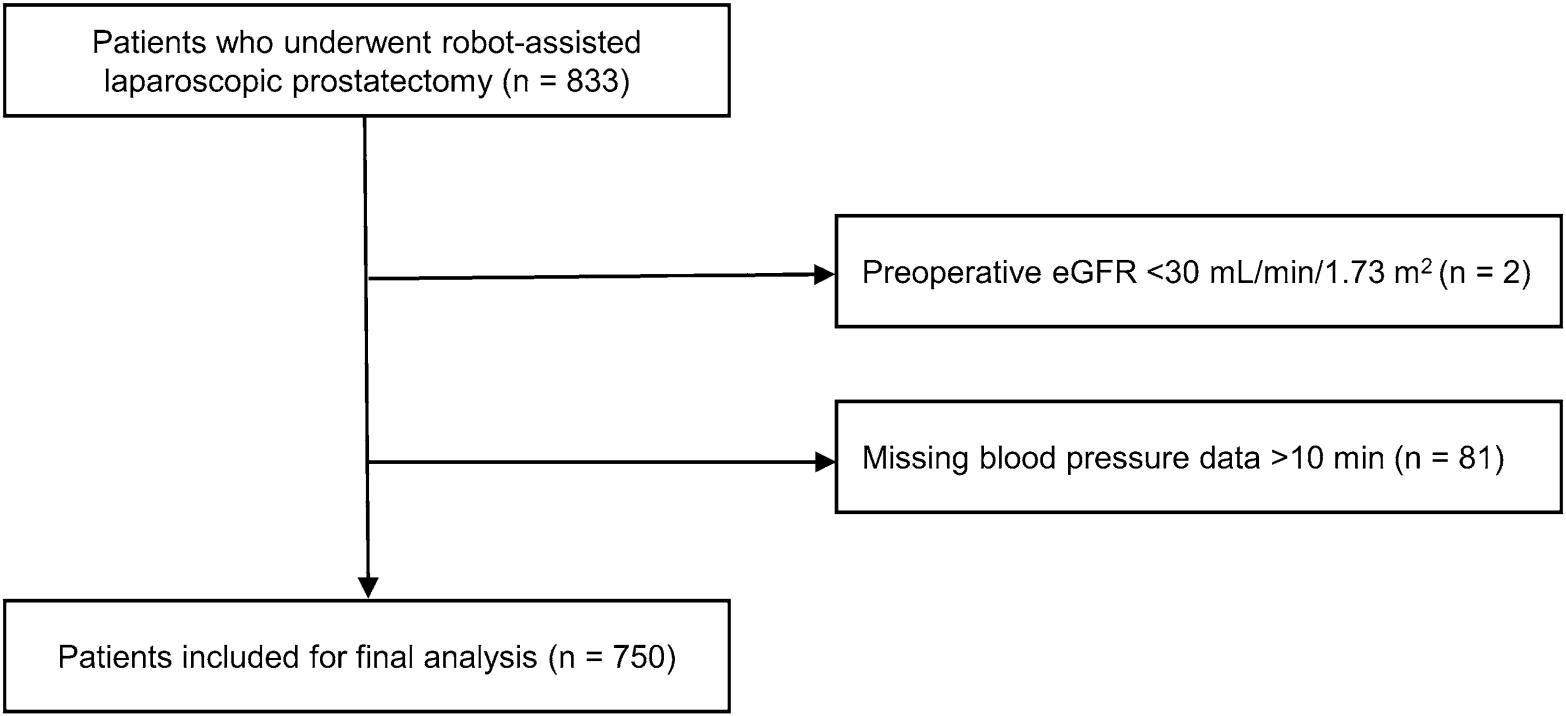
Flow chart of the study population.

Among 833 screened patients, we excluded two patients with a preoperative estimated glomerular filtration rate (eGFR) < 30 mL/min/1.73 m^2^ and 81 patients with missing intraoperative BP for > 10 min. Finally, we included 750 patients (Fig. 1). Out of the 750 patients, 18 (2.4%) patients developed postoperative AKI. Table 1 summarizes the patient characteristics. Patients who developed AKI had a significantly higher American Society of Anesthesiologists (ASA) physical status, and were more likely to have diabetes mellitus, coronary artery disease, and atrial fibrillation than those who did not develop AKI. Additionally, a significantly greater proportion of patients with AKI was taking angiotensin-converting enzyme inhibitor (ACEI)/angiotensin receptor blocker (ARB) or diuretics than those without AKI. Table 2 summarizes the intraoperative characteristics. Patients with AKI had significantly longer Trendelenburg position time, lesser amount of urine output, and greater number of transfusions compared with those without AKI.

**Table 1:** Patient characteristics.

Variables	All (n = 750)	No AKI (n = 732)	AKI (n = 18)	*P* value
Age, y	68 [63–73]	68 [63–73]	71 [63–75]	0.438
Body mass index, kg/m^2^	25.0 [23.1–26.7]	25.0 [23.1–26.7]	26.4 [24.4–28.1]	0.084
ASA Physical status				< 0.001
I	117 (15.6%)	116 (15.9%)	1 (5.6%)	
II	402 (53.6%)	398 (54.4%)	4 (22.2%)	
III	231 (30.8%)	218 (29.8%)	13 (72.2%)	
Comorbidities				
Hypertension	428 (57.1%)	414 (56.6%)	14 (77.8%)	0.072
Diabetes mellitus	133 (17.7%)	126 (17.2%)	7 (38.9%)	0.027
Coronary artery disease	50 (6.7%)	46 (6.3%)	4 (22.2%)	0.027
COPD	67 (8.9%)	65 (8.9%)	2 (11.1%)	0.671
Atrial fibrillation	27 (3.6%)	24 (3.3%)	3 (16.7%)	0.024
Chronic kidney disease	21 (2.8%)	19 (2.6%)	2 (11.1%)	0.087
Preoperative medication				
Calcium channel blocker	249 (33.2%)	243 (33.2%)	6 (33.3%)	0.990
β-blocker	67 (8.9%)	65 (8.9%)	2 (11.1%)	0.671
ACEI or ARB	321 (42.8%)	308 (42.1%)	13 (72.2%)	0.011
Diuretics	80 (10.7%)	75 (10.3%)	5 (27.8%)	0.034
Preoperative laboratory values				
Hematocrit, %	42.1 [39.6–44.3]	42.1 [39.6–44.3]	43.2 [39.6–44.6]	0.427
Serum albumin, g/dL	4.6 [4.4–4.8]	4.6 [4.4–4.8]	4.6 [4.4–4.7]	0.481
Serum sodium, mmol/L	140 [139–142]	140 [139–142]	141 [139–141]	0.849
eGFR, mL/min/1.73 m^2^	86 [77–93]	86 [77–93]	78 [56–91]	0.081
Preoperative MAP, mmHg	97 [91–103]	97 [91–103]	97 [92–104]	0.888

Values are median (interquartile rage) or number of patients (percentage).

*AKI* acute kidney injury, *ASA* American Society of Anesthesiology, *ACEI* angiotensin-converting enzyme inhibitor, *ARB* angiotensin receptor blocker, *COPD* chronic obstructive pulmonary disease, *eGFR* estimated glomerular filtration rate, *MAP* mean arterial pressure.

**Table 2:** Intraoperative characteristics.

Variables	All (n = 750)	No AKI (n = 732)	AKI (n = 18)	*P* value
Anesthesia time, min	140 [125–160]	140 [125–160]	153 [120–185]	0.497
Trendelenburg position time, min	57 [50–74]	57 [49–73]	76 [56–96]	0.013
Fluid intake, mL	1100 [910–1300]	1100 [905–1300]	1200 [1000–1600]	0.066
Urine output, mL	200 [100–250]	200 [100–250]	115 [100–200]	0.028
Blood loss, mL	300 [200–500]	300 [200–500]	400 [200–550]	0.222
Transfusion of red blood cells, yes	5 (0.7%)	3 (0.4%)	2 (11.1%)	0.005
Administered dose of ephedrine, mg	4 (0–8)	4 (0–8)	4 (0–8)	0.391
Use of NE or Phenyl, yes	49 (6.5%)	48 (6.6%)	1 (5.6%)	> .999
Administered dose of remifentanil, µg	386 [304–500]	385 [304–500]	471 [330–612]	0.077

Values are median (interquartile rage) or number of patients (percentage).

*AKI* acute kidney injury, *NE* norepinephrine, *Phenyl* phenylephrine.

### Mean arterial pressure (MAP) parameters

**Figure 2:**
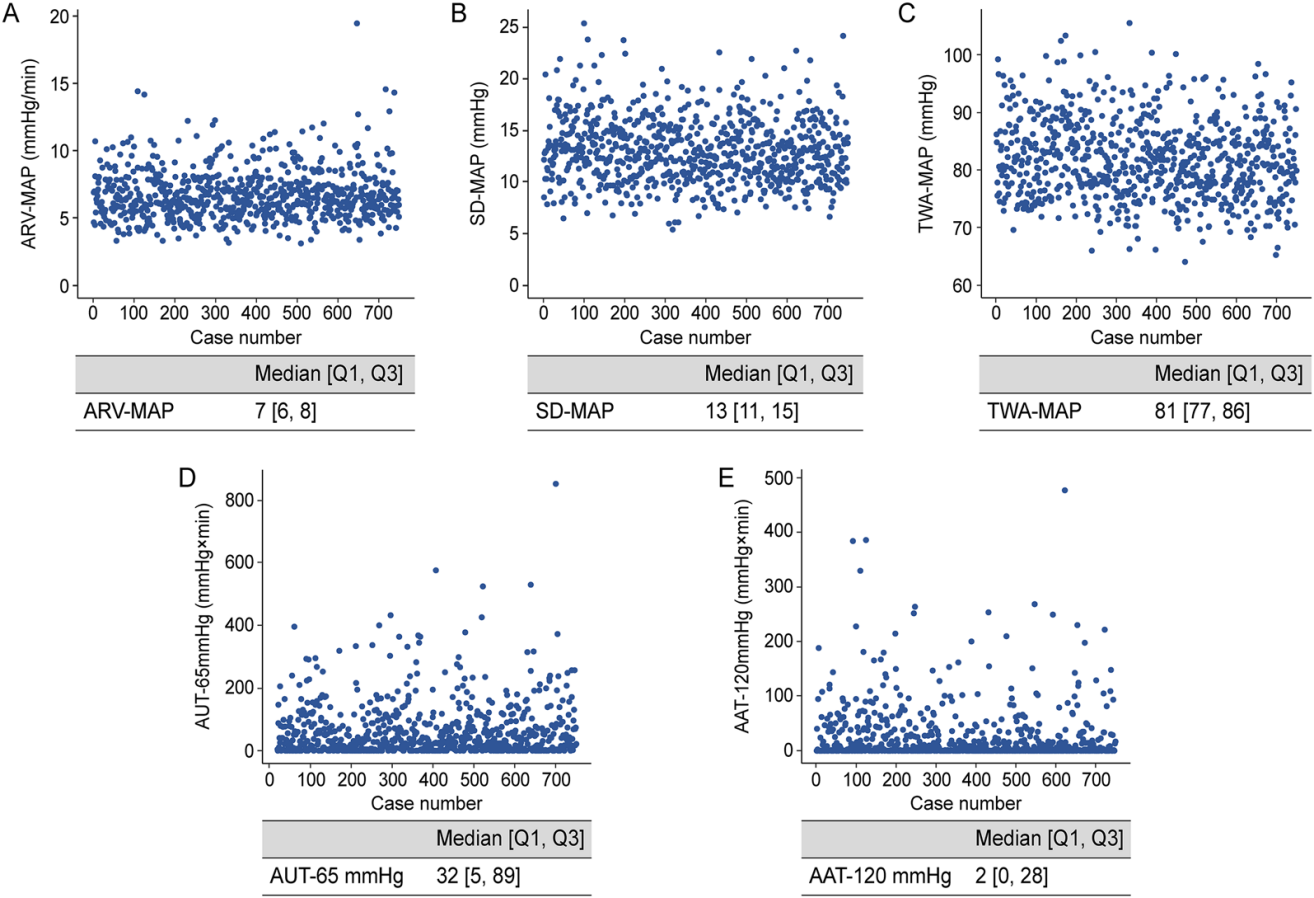
Scatterplots of the mean arterial pressure parameters. The distribution of the mean arterial pressure parameters: (**A**) average real variability of mean arterial pressure (ARV-MAP), (**B**) standard deviation of mean arterial pressure (SD-MAP), (**C**) time-weighted average of mean arterial pressure (TWA-MAP), (**D**) area under threshold of mean arterial pressure of 65 mmHg (AUT-65 mmHg), and (**E**) area above threshold of mean arterial pressure of 120 mmHg (AAT-120 mmHg) within the study population. Q1, first quartile; Q3, third quartile.

**Figure 3:**
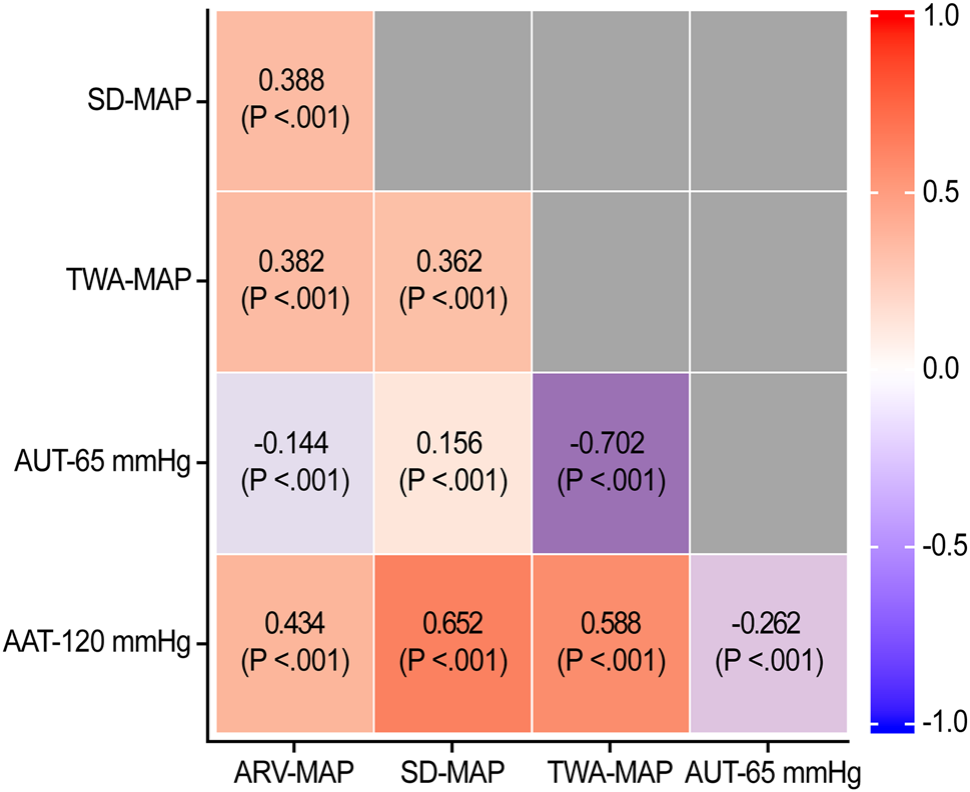
Heat map showing the correlations among mean arterial pressure parameters. The squares show the correlation coefficients (− 1 to 1), with *P* values, between the variables according to row and column. The red and blue show positive and negative correlations, respectively. ARV, average real variability; SD, standard deviation; TWA, time-weighted average; MAP, mean arterial pressure; AUT, area under threshold; AAT, area above threshold.

Among the 750 included patients, 642,336 measurements of continuous MAP were obtained. The median [IQR] number of measured MAPs per surgery was 817 [728–930]. Figure 2 shows the distributions of MAP parameters. The median [IQR] values of the average real variability (ARV)-MAP, standard deviation (SD)-MAP, time-weighted average (TWA)-MAP, area under threshold (AUT)-65 mmHg, and area above threshold (AAT)-120 mmHg were 7 [6–8] mmHg/min, 13 [11–15] mmHg, 81 [77–86] mmHg, 32 [5–89] mmHg × min, and 2 [0–28] mmHg × min, respectively. There were significant correlations among MAP parameters (Fig. 3). ARV-MAP was positively correlated with all other MAP parameters except AUT-65 mmHg (Fig. 3). Body mass index (BMI) and preoperative MAP showed a significant positive correlation with ARV-MAP; whereas, patients with diabetes mellitus had significantly lower ARV-MAP values (Table 3).

**Table 3:** Correlation between average real variability of mean arterial pressure and clinical factors.

Variables	Coefficient	*P* value
Age	− 0.069	0.060
Body mass index	0.083	0.022
ASA physical status	− 0.009	0.809
Hypertension	− 0.030	0.408
Diabetes mellitus	− 0.081	0.026
ACEI or ARB	− 0.026	0.479
Preoperative MAP	0.158	< 0.001

*ASA* American Society of Anesthesiology, *ACEI* angiotensin-converting enzyme inhibitor, *ARB* angiotensin receptor blocker, *MAP* mean arterial pressure.

### Primary and secondary outcomes

Among the 18 patients who developed AKI, 12 (66.7%) patients, 3 (16.7%) patients, and 3 (16.7%) patients were stage 1, 2, and 3, respectively. In the univariable analysis of risk factors for AKI, 19 factors including TWA-MAP and AUT-65 mmHg revealed *P* < 0.2 (Table 4). Out of these 19 factors, hypertension, diabetes mellitus, coronary artery disease, atrial fibrillation, and chronic kidney disease were excluded in the multivariable analysis because they are strongly related to ASA physical status. Moreover, exclusion of chronic kidney disease can be rationalized due to the selection of preoperative eGFR. The ‘Trendelenburg position time’ and the ‘anesthesia time’ were highly correlated (correlation coefficient = 0.84), thus we chose the ‘Trendelenburg position time’ due to its lower *P* value compared to the ‘anesthesia time’ (*P* = 0.004 vs *P* = 0.107). Finally, 13 factors were included in the multivariable analysis. ASA physical status ≥ III (odd ratio [OR] = 4.97, 95% confidence interval [CI] 1.51–16.34) and the intraoperative urine output (OR = 0.61, 95% CI 0.42–0.89 per 50 mL increase) were independently associated with the occurrence of AKI (Table 4).

**Table 4:** Univariable and multivariable analyses of risk factors for postoperative acute kidney injury.

	Univariable			Multivariable	
	OR (95% CI)	*P* value		OR (95% CI)	*P* value
ARV-MAP, 10 mmHg/min increase	2.94 (0.32–27.1)	0.342			
SD-MAP, 10 mmHg increase	1.58 (0.38–6.63)	0.534			
TWA-MAP, 10 mmHg increase	1.55 (0.81–2.99)	0.189		1.71 (0.61–4.77)	0.310
AUT-65 mmHg, 50 mmHg × min increase	0.70 (0.44–1.13)	0.146		0.62 (0.35–1.09)	0.095
AAT-120 mmHg, 50 mmHg × min increase	1.11 (0.76–1.61)	0.600			
Age, 10 y increase	1.29 (0.65–2.55)	0.465			
Body mass index, 5 kg/m^2^ increase	2.05 (0.94–4.49)	0.073		1.87 (0.68–5.09)	0.224
ASA physical status ≥ III (referent I & II)	6.13 (2.16–17.4)	0.001		4.97 (1.51–16.3)	0.008
Comorbidities					
Hypertension	2.69 (0.88–8.25)	0.084			
Diabetes mellitus	3.06 (1.16–8.05)	0.023			
Coronary artery disease	4.26 (1.35–13.5)	0.014			
COPD	1.28 (0.29–5.70)	0.743			
Atrial fibrillation	5.90 (1.60–21.8)	0.008			
Chronic kidney disease	4.69 (1.01–21.9)	0.049			
Preoperative medication					
β-blocker	1.28 (0.29–5.70)	0.743			
Calcium channel blocker	1.01 (0.37–2.71)	0.990			
ACEI or ARB	3.58 (1.26–10.14)	0.016		2.79 (0.78–9.96)	0.115
Diuretics	3.37 (1.17–9.71)	0.025		1.67 (0.47–5.93)	0.432
Preoperative MAP, 10 mmHg increase	0.92 (0.53–1.62)	0.777			
Preoperative laboratory values					
Hematocrit, 3% increase	1.18 (0.79–1.77)	0.427			
Serum albumin, 1 g/dL increase	0.62 (0.12–3.30)	0.571			
eGFR, 10 mL/min/1.73 m^2^ increase	0.67 (0.50–0.89)	0.007		0.75 (0.54–1.05)	0.094
Serum sodium, 5 mmol/L increase	1.15 (0.37–3.59)	0.814			
Trendelenburg position time, 10 min increase	1.17 (1.05–1.29)	0.004		1.01 (0.80–1.28)	0.936
Anesthesia time, 30 min increase	1.28 (0.95–1.73)	0.107			
Fluid intake, 100 mL increase	1.14 (1.04–1.26)	0.007		1.27 (0.99–1.62)	0.058
Urine output, 50 mL increase	0.74 (0.56–0.99)	0.039		0.61 (0.42–0.89)	0.009
Blood loss, 100 mL increase	1.13 (1.00–1.28)	0.059		0.77 (0.58–1.02)	0.065
Transfusion of red blood cells, yes	30.4 (4.75–194)	< .001		22.4 (0.51–988)	0.107
Administered dose of remifentanil, 100 µg increase	1.29 (1.05–1.60)	0.016		1.13 (0.77–1.66)	0.522
Administered dose of ephedrine, 4 mg increase	0.91 (0.67–1.23)	0.526			
Use of NE or phenyl, yes	0.84 (0.11–6.43)	0.865			

*OR* odds ratio, *CI* confidence interval, *ARV* average real variability, *SD* standard deviation, *TWA* time-weighted average, *MAP* mean arterial pressure, *AUT* area under threshold, *AAT* area above threshold, *ASA* American Society of Anesthesiologists, *COPD* chronic obstructive pulmonary diseas, *ACEI* angiotensin-converting enzyme inhibitor, *ARB* angiotensin receptor blocker, *eGFR* estimated glomerular filtration rate, *NE* norepinephrine, *Phenyl* phenylephrine.

Table 5 presents the area under the receiver operating characteristic curve (AUROC) values of each MAP parameter for predicting AKI after RALP. No MAP parameter could predict postoperative AKI. Specifically, the AUROC values for ARV-MAP, SD-MAP, TWA-MAP, AUT-65 mmHg, and AAT-120 mmHg were 0.561 (95% confidence interval [CI], 0.424–0.697), 0.561 (95% CI, 0.417–0.704), 0.584 (95% CI, 0.458–0.709), 0.590 (95% CI, 0.462–0.718), and 0.626 (95% CI, 0.499–0.753), respectively.

**Table 5:** Area under the receiver operating characteristic curve of each mean arterial pressure parameter for predicting postoperative acute kidney injury.

Parameters	Area under the curve	95% CI	*P* value
ARV-MAP, mmHg/min	0.561	0.424–0.697	0.342
SD-MAP, mmHg	0.561	0.417–0.704	0.534
TWA-MAP, mmHg	0.584	0.458–0.709	0.189
AUT-65 mmHg, mmHg × min	0.590	0.462–0.718	0.146
AAT-120 mmHg, mmHg × min	0.626	0.499–0.753	0.325

*CI* confidence interval, *ARV* average real variability, *SD* standard deviation, *TWA* time-weighted average, *MAP* mean arterial pressure, *AUT* area under threshold, *AAT* area above threshold.

## Discussion

This is the first retrospective study to explore the relationship between intraoperative MAP and AKI occurrence after RALP. Pneumoperitoneum and the steep Trendelenburg position during RALP cause diverse intraoperative MAP changes. However, we observed no association between these changes (MAP variability, hypotension, and hypertension) and AKI after RALP. In contrast, ASA physical status ≥ III and the low intraoperative urine output were independently associated with the occurrence of AKI.

Pneumoperitoneum, which is dependent on the amount of intra-abdominal pressure, can induce direct renal vascular and parenchymal compression as well as the release of antidiuretic hormone, renin, and aldosterone, which results in decreased renal blood flow, GFR, and renal excretory function^3,4^. However, it remains unclear whether compared with open radical prostatectomy, RALP increases the risk of AKI. A study reported that compared with open radical prostatectomy, RALP involves a significantly lower incidence of AKI^5^. However, in another study, 25 (13.4%) out of 187 patients who underwent RALP showed an acute increase in SCr levels on the operation day, which met the Kidney Disease Improving Global Outcomes (KDIGO) criteria for AKI; however, none of the patients who underwent open radical prostatectomy met this criteria^7^. Therefore, AKI after RALP remains a concern; accordingly, there have been clinical trials for mitigating AKI in patients undergoing RALP. Intraoperative infusion of low-dose (0.5 µg/kg/min) nicardipine, which is a calcium channel blocker, has been found to improve renal function on postoperative day 1^19^. Contrastingly, intraoperative infusion of mannitol (0.5 g/kg) did not facilitate the prevention of AKI after RALP^6^.

Numerous factors, including CO_2_ gas insufflation and desufflation as well as performing a steep Trendelenburg position and resuming a supine position, induce abrupt changes in BP during RALP^9–12^. A large-scale study on patients undergoing non-cardiac surgery reported a positive correlation of intraoperative MAP variability with the risk of postoperative AKI, regardless of intraoperative hypotension^13^. Similarly, systolic BP variability is negatively correlated with renal function in patients with hypertension^20,21^. Renal perfusion is maintained by neurohormonal responses over time^19,22^; therefore, abrupt BP fluctuations may exceed the capacity of such adaptations, which may result in kidney damage. However, we found that ARV-MAP was not related to and a poor predictor of AKI after RALP (Tables 4 and 5). The median [IQR] ARV-MAP was 7 [6–8] mmHg/min, with the lowest and highest values being 3 and 19 mmHg/min, respectively (Fig. 2). Therefore, the BP variability during RALP may be tolerable with respect to renal function.

There remains no recommended standard measurement for BP variability. We assessed MAP variability using the SD-MAP and ARV-MAP. ARV-MAP may be more appropriate than SD-MAP since ARV represent consecutive changes in MAP, while SD does not consider the timing of measurements^13,18^. Since we preferred ARV-MAP over SD-MAP, we identified preoperative factors correlated with ARV-MAP (Table 3). BMI was positively correlated with intraoperative ARV-MAP, which is consistent with findings from previous reports that showed that compared with normal-weight patients, overweight and obese patients present higher BP variability during their daily lives^23,24^. We found that patients with diabetes mellitus showed low ARV-MAP, which is inconsistent with the results of a previous report of high intraoperative BP variability in patients with diabetes mellitus^13^. Although hypertension and treatment with ACEI or ARB did not affect ARV-MAP, intraoperative ARV-MAP was positively correlated with preoperative MAP. Additionally, ARV-MAP showed a positive correlation with TWA-MAP and AUT-120 mmHg as well as a negative correlation with AUT-65 mmHg (Fig. 3). These findings suggest a positive correlation of BP with BP variability, which is consistent with a previous finding of high BP variability in patients with uncontrolled hypertension^25^.

Intraoperative hypotension is known to be strongly correlated with AKI after non-cardiac surgery^13–17^. Absolute and relative (reduction from baseline) MAP thresholds have been used to define hypotension^14,15,26^. However, the association of relative and absolute hypotension thresholds with AKI have similar strengths^15^. Absolute thresholds are easier to use in decision-making without requiring preoperative BP data, with an absolute MAP threshold of < 65 mmHg being the most commonly used^14,26^. Therefore, we used a MAP threshold of < 65 mmHg and calculated AUT-65 mmHg, which characterizes the hypotension duration and severity (amount of hypotension). However, AUT-65 mmHg was not associated with and a poor predictor of AKI after RALP (Tables 4 and 5), which could be attributed to our low incidence of AKI. A previous study on 138,021 non-cardiac surgeries demonstrated that the relationship of intraoperative hypotension with AKI varied according to the underlying patient and procedural risks. Specifically, intraoperative hypotension was associated with AKI in patients with medium and high but not low risk^27^. The AKI incidence was 1.7% and ≥ 4.6% in patients with low and medium risk, respectively^27^. In our study, the AKI incidence was 2.4%, which suggests that patients undergoing RALP had a low risk of AKI and that intraoperative hypotension is not an important determinant of AKI. However, further studies are warranted to elucidate the impact of intraoperative hypotension on AKI after RALP in high-risk patients. Alternatively, TWA-MAP, which represent the overall BP and AAT-120 mmHg, indicating the hypertension duration and severity, were also not associated with and not good predictors of AKI after RALP (Tables 4 and 5). Therefore, the high BP during RALP might be tolerable and not cause renal damage. Although the association of high BP with increased postoperative morbidity remains unclear, compared with hypotension, elevated intraoperative BP may not be as strongly associated with postoperative morbidity^26^.

In our study, ASA physical status ≥ III and the low intraoperative urine output were revealed to be independent predictors of postoperative AKI. After adjusting for confounding factors, patients with an ASA physical status ≥ III had an approximately fivefold increased risk of AKI compared to the ones with an ASA physical status I or II. Consistent with our result, ASA physical status was proven as a determinant for postoperative AKI in non-cardiac surgery^28,29^ and an ASA physical status ≥ III showed an approximately twofold increased risk of AKI^28^. The predictive value of intraoperative urine output for postoperative AKI remains controversial. There are some studies stating that there is no association between intraoperative urine output and the occurrence of AKI^30,31^, while others are advocating for the association between oliguria and the occurrence of AKI after major non-cardiac surgery^28,32,33^. Although some previous studies investigated the risk factors for AKI after RALP, none of those included the urine output as a candidate variable^6–8^. Therefore, our study is the first to identify the low intraoperative urine output as a risk factor for AKI in RALP. However, future studies investigating whether increasing the urine output with the use of diuretics would prevent the development of AKI after RALP are needed to confirm the causative effect of intraoperative urine output on AKI after RALP.

Our findings have strength in terms of the elaborate data quality. Specifically, the MAP values were obtained through invasive arterial monitoring and collected in 10-s intervals; accordingly, 642,336 continuous MAP measurements were included in the analysis. Nevertheless, this study has several limitations. First, this was a single-center retrospective study, which increases the risk of bias and the influence of confounding factors. Although we adjusted for confounding factors based on a previous report^34^, there could have been unknown and unadjusted confounding factors. Second, we did not consider the use of vasopressors or vasodilators given their dose complexity and inaccuracies. Further studies that consider BP-modifying drugs are warranted to validate our findings. Finally, the lower incidence of AKI in our study, may have led to the conclusion of no association of any MAP parameters with AKI. The AKI incidence in our study was 2.4%, which is half of the previously reported incidence (5%) in RALP^5,6^. Some studies have demonstrated that a longer operation time represents a risk factor for AKI after RALP^6–8^. Therefore, our low AKI incidence could be attributed to shorter operation times than those observed in previous studies in which the mean operation time was around 170–180 min^6^. Therefore, multi-center large-scale trials are needed to confirm the association between MAP parameters and AKI after RALP.

In conclusion, ASA physical status ≥ III and the low intraoperative urine output were independent risk factors for AKI after RALP. At the levels observed in the present study, we were unable to demonstrate an association between intraoperative MAP changes, including MAP variability, hypotension, and hypertension, and AKI after RALP. Therefore, dynamic BP changes during RALP may be within the acceptable range for kidney perfusion; accordingly, intraoperative MAP changes may not be an important determinant of postoperative AKI.

## Materials and methods

### Selection of participants

This single-center retrospective study was approved by the Institutional Review Board and Hospital Research Ethics Committee of Severance Hospital, Yonsei University Health System, Seoul, Korea (number: 4–2021-0434, approved on 25 May 2021) and was performed in accordance with relevant guidelines and regulations. The requirement for informed consent was waived by the Severance hospital ethical committee's institutional review board given the retrospective nature of the study. We screened adult patients who underwent RALP between January 2020 and April 2021; among them, we excluded patients with preoperative eGFR < 30 mL/min/1.73 m^2^ and those who had missing intraoperative BP for > 10 min.

### Data collection

We retrospectively collected the following demographic characteristics from the patients’ electronic medical records: age, BMI, ASA physical status, and comorbidities such as hypertension (previously diagnosed and currently taking antihypertensive medications), diabetes mellitus (previously diagnosed and taking antidiabetic medications), coronary artery disease (history of coronary angioplasty and stent insertion), chronic obstructive pulmonary disease (emphysema or chronic bronchitis under use of bronchodilators), atrial fibrillation (confirmed by preoperative electrocardiogram), and chronic kidney disease (eGFR < 60 ml/min/1.73 m^2^ for > 3 months).

Moreover, we collected preoperative medication data, including the use of calcium channel blocker, β-blocker, ACEI or ARB, and diuretics, as well as preoperative laboratory data, including hematocrit, serum albumin, serum sodium levels, and eGFR, which are established predictors for AKI^34^. The eGFR was calculated based on SCr levels using the Chronic Kidney Disease Epidemiology Collaboration Eq. ^35^. Regarding preoperative MAP, we calculated the average of two MAPs calculated as follows: MAP = diastolic BP + 1/3 [systolic BP–diastolic BP] based on BP measurements obtained twice preoperatively. We collected the following intraoperative information: anesthesia duration, duration of Trendelenburg position, fluid intake volume, urine output, blood loss, transfusion of red blood cells (yes/no), administered dose of remifentanil and ephedrine, and use of norepinephrine/phenylephrine (yes/no). Postoperative SCr levels were measured within the first 7 postoperative days.

### Intraoperative blood pressure

Noninvasive BP measurements were obtained using BP cuffs at 1- to 5-min intervals during the anesthesia-induction periods. After anesthesia induction, all patients underwent radial arterial cannulation with a 20-gauge catheter. Recordings of noninvasive and invasive BPs were automatically saved in the VitalDB database via the VitalRecorder, which is a recent software for querying large databases containing high-resolution time-synchronized physiological data obtained using multiple anesthesia devices (https://vitaldb.net)^36,37^. We downloaded raw data obtained at 10-s intervals and removed artifacts based on the following respective criteria: (1) only systolic or diastolic BP was measured, (2) the systolic BP was > 10 times the diastolic BP, (3) systolic BP > 300 mmHg or diastolic BP > 200 mmHg, (4) systolic BP < 30 mmHg or diastolic BP < 20 mmHg, and (5) diastolic BP was > 95% of the systolic BP^13^.

### Mean arterial pressure parameters

We calculated five MAP parameters using the measured MAP values. ARV-MAP (mmHg/min) and SD-MAP (mmHg) were used as measures of MAP variability^18^. The ARV-MAP and SD-MAP were calculated using the following equations:

$${\text{ARV}} - {\text{MAP}} = \frac{1}{{\text{T}}}\mathop \sum \limits_{{{\text{i}} = 1}}^{{{\text{N}} - 1}} \left| {{\text{X}}_{{{\text{i}} + 1}} - {\text{X}}_{{\text{i}}} } \right|,{\text{SD}} - {\text{MAP}} = \sqrt {\frac{{\mathop \sum \nolimits_{{{\text{i}} = 1}}^{{\text{N}}} \left( {{\text{x}}_{{\text{i}}} - {\overline{\text{x}}}} \right)^{2} }}{{\left( {{\text{N}} - 1} \right)}}}$$

N, T, and ${\overline{\text{x}}}$ represent the number of BP readings, total time (min) from first to last BP reading, and mean MAP value, respectively.

TWA-MAP (mmHg) was calculated as the area under the curve value of the MAP measurements divided by the total measurement time for the mean MAP. AUT-65 mmHg was calculated as the area under a MAP of 65 mmHg while AAT-120 mmHg was calculated as the area above a MAP of 120 mmHg. AUT-65 mmHg (mmHg × min) and AAT-120 mmHg (mmHg × min) present the duration and severity of hypotension and hypertension, respectively^26^.

### Study outcome

The primary endpoint was the association of intraoperative MAP (MAP variability, hypotension, and hypertension) with AKI after RALP. The secondary endpoint was the association of other factors with AKI after RALP. AKI occurrence was based on the KDIGO criteria, i.e., an increase in the SCr level by ≥ 0.3 mg/dL within 48 h or an increase in the SCr level to ≥ 1.5 times the baseline value within 7 postoperative days^38^. AKI was further categorized into the following three stages: stage 1, an increase in SCr level by 1.5–1.9 times baseline or by ≥ 0.3 mg/dL; stage 2, an increase in SCr level by 2.0–2.9 times baseline; stage 3, an increase in SCr level by at least 3.0 times baseline or to ≥ 4.0 mg/dL, or the initiation of renal replacement therapy^38^.

### Statistical analysis

Continuous and categorical variables are presented as median [IQR] and number (percentage), respectively. Spearman’s correlation coefficients were used to determine the correlations among MAP parameters and the correlations of ARV-MAP with clinical factors. A binary logistic regression analysis was performed to find risk factors for the occurrence of AKI. Variables with *P* < 0.2 in the univariable analysis were included in the multivariable logistic regression model. However, in order to avoid multicollinearity, only one variable was chosen from a group of highly correlated continuous variables, and the same applies to a group of highly related categorical variables. Receiver operating characteristic curve analysis was performed and the AUROC value was calculated to evaluate the discrimination ability of each MAP parameter to predict AKI. Statistical significance was set at *P* < 0.05; moreover, all tests were two-tailed. Statistical analyses were performed using SAS version 9.4 (SAS Institute) and R version 4.0.4 (R Project for Statistical Computing).

## Data Availability

The datasets used during the current study are available from the corresponding author on reasonable request.
